# Occlusive dressing therapy using dimethyl sulfoxide in a patient presenting with primary localized amyloidosis of the urinary bladder: a case report

**DOI:** 10.1186/1752-1947-7-191

**Published:** 2013-07-26

**Authors:** Tateki Yoshino, Shinya Ohara, Hiroyuki Moriyama

**Affiliations:** 1 Department of Urology, JA Onomichi General Hospital, 1-10-23 Hirahara, Onomichi, Hiroshima, 722-0018, Japan; 2 Department of Urology, Integrated Health Sciences, Institute of Biochemical & Health Sciences, Hiroshima University, Hiroshima, 734-8551, Japan

**Keywords:** Amyloidosis, Dimethyl sulfoxide, Occlusive dressing therapy

## Abstract

**Introduction:**

Amyloidosis is characterized by extracellular deposition of abnormal insoluble fibrils, which cause structural and functional disorders. Amyloidosis is classified into primary and secondary disease. We report a case of localized amyloidosis of the urinary bladder. In the English literature, this is the first case effectively treated with occlusive dressing therapy using dimethyl sulfoxide.

**Case presentation:**

A 58-year-old Japanese woman was introduced to our department with asymptomatic gross hematuria. Cystoscopy revealed a gently raised nodule at the right lateral wall. Histopathological findings of this lesion revealed extensive amorphous eosinophilic deposits that stained positive with Congo red and Dylon. The patient was diagnosed with primary localized amyloidosis of the urinary bladder. To treat residual amyloidosis of the bladder, we performed occlusive dressing therapy using dimethyl sulfoxide. After treatment, cystoscopy and magnetic resonance imaging showed no relapse of the mass-like lesion of the bladder wall.

**Conclusions:**

Occlusive dressing therapy using dimethyl sulfoxide is efficacious and tolerable for amyloidosis of the urinary bladder. The maneuver of occlusive dressing therapy was simpler and easier than that of intravesical instillation, and occlusive dressing therapy was advantageous in that the patient could perform the therapy herself every day. However, invasive surgical management including cystectomy should be considered if conservative management is inefficacious.

## Introduction

Amyloidosis is a heterogenous group of diseases that share the common feature of extracellular deposition of an insoluble fibrillar protein in organs and tissues. Amyloidosis can also be classified as primary or secondary disease. The primary type (AL amyloidosis) is associated with an underlying immune dyscrasia, including multiple myeloma or Waldenström’s macroglobulinemia. In the secondary type (AA amyloidosis), an underlying chronic inflammatory disease often arises as a complication. Primary localized amyloidosis of the urinary bladder is a rare condition with approximately 200 reported cases in the literature [1-3]. The clinical presentation and cystoscopic appearance of this condition are known to mimic bladder cancer.

## Case presentation

**Figure 1:**
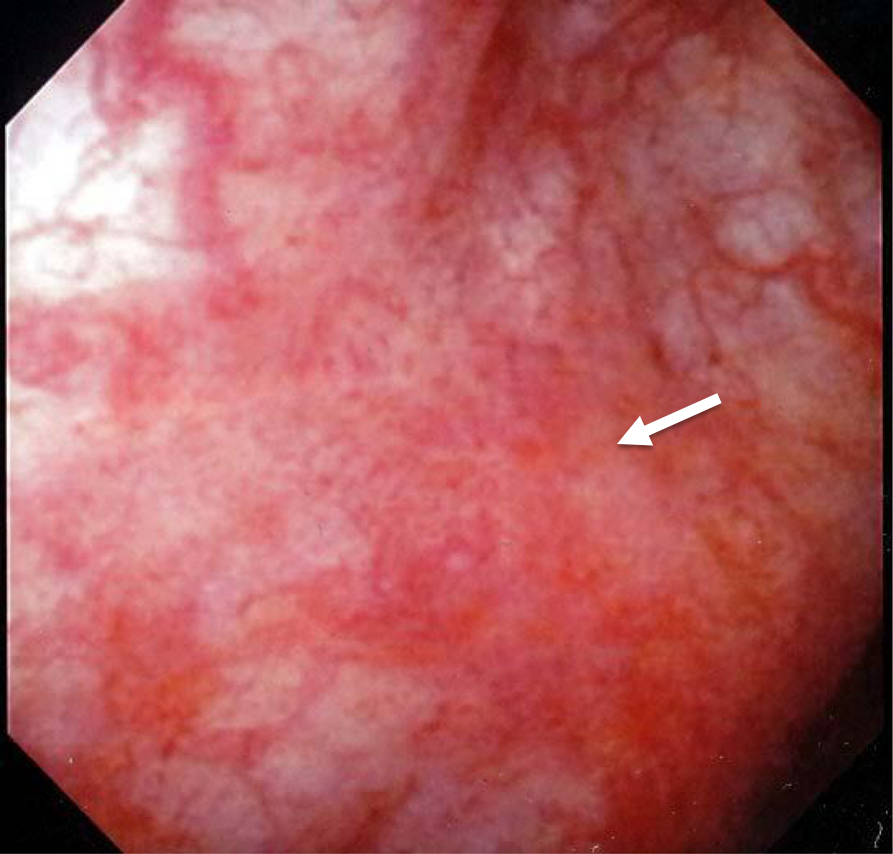
Cystoscopic findings showed a gently raised nodule with erythema at the right lateral wall (arrows).

**Figure 2:**
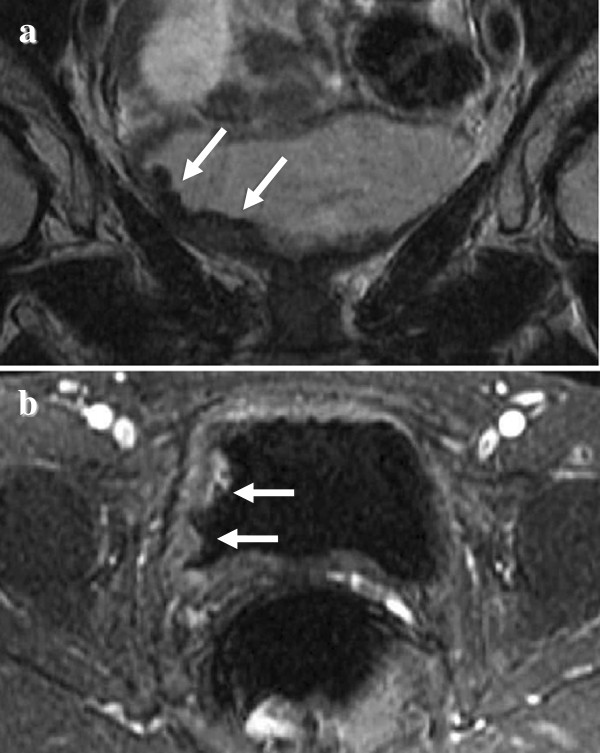
**Magnetic resonance imaging findings of the pelvis (a: coronal section, b: transverse section).** Magnetic resonance imaging indicated thickness of the right lateral wall of the urinary bladder (arrow).

A 58-year-old Japanese woman was introduced to our department with asymptomatic gross hematuria. Her past medical history was unremarkable. There was no history of urinary symptoms and urinary tract infection. The results of a physical examination were unremarkable. Urinalysis showed microscopic hematuria and no pyuria. Urine cytology indicated atypical cells. A cystoscopy revealed a gently raised nodule with erythema at the right lateral wall (Figure 1). Contrast-enhanced magnetic resonance imaging (MRI) at 3.0 tesla also indicated thickness of the right lateral wall (Figure 2), which was suspected for muscle invasion of bladder cancer.

**Figure 3:**
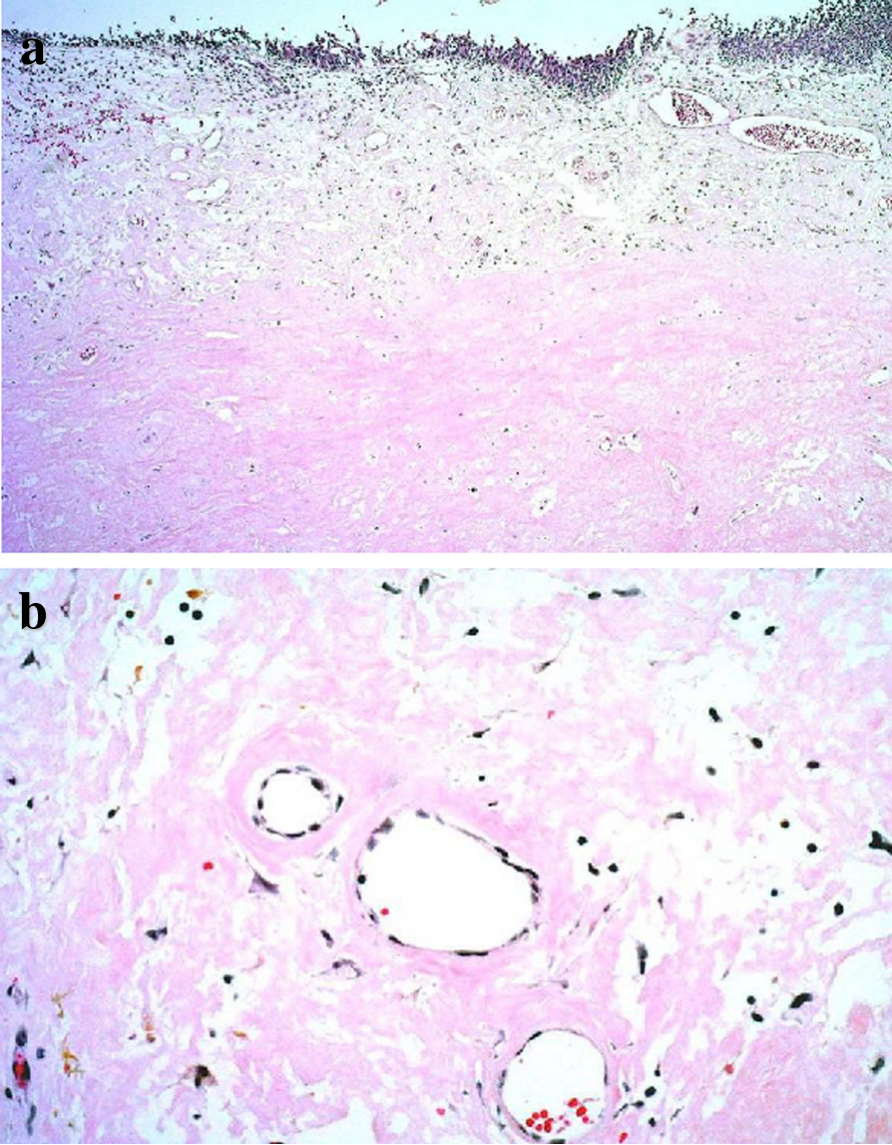
**Histopathological findings of the specimens revealed extensive extracellular amorphous eosinophilic deposits in the subepithelial layer.** Hematoxylin and eosin stain, **a**: ×40, **b**: ×200.

**Figure 4:**
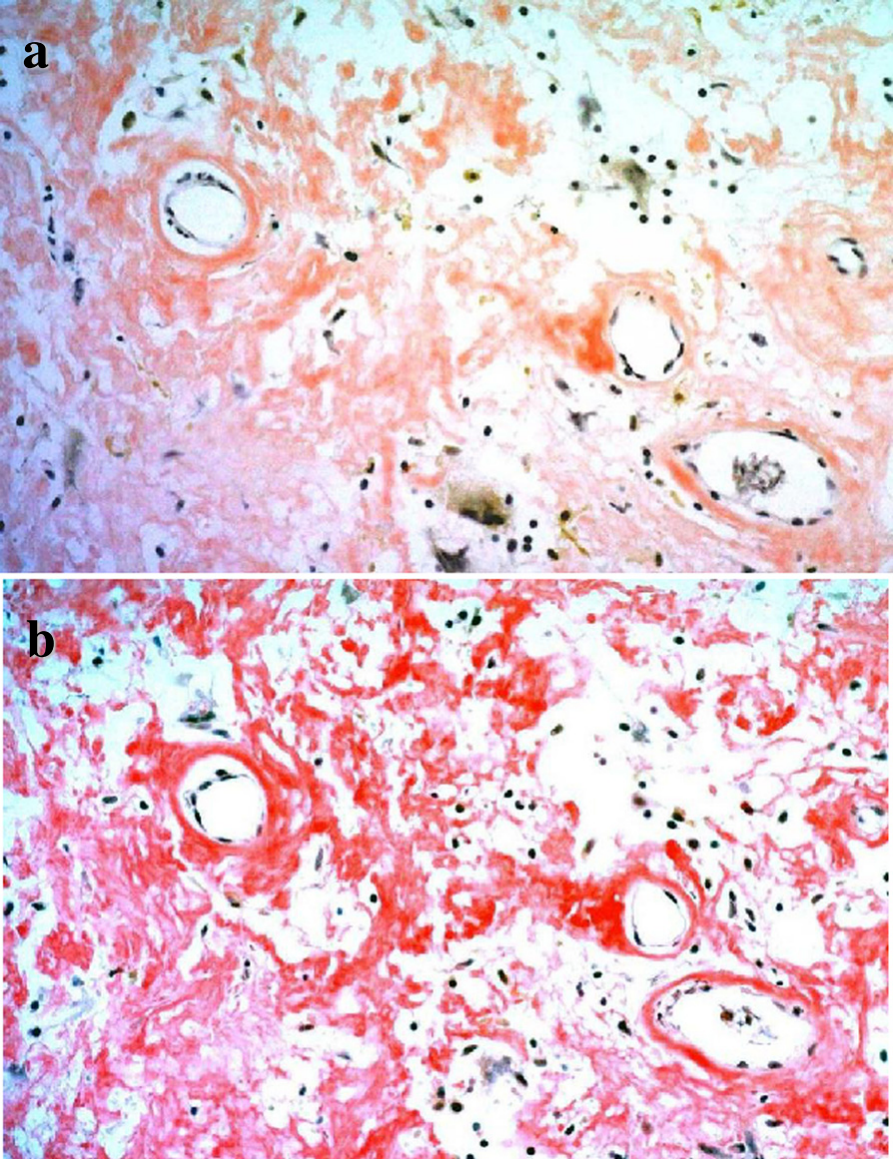
**Immunohistochemical analysis showed positive staining at eosinophilic deposits. a**: Congo red stain ×200, **b**: Dylon stain ×200.

The patient underwent transurethral resection of the bladder lesions. Histopathological findings of all the specimens revealed extensive extracellular amorphous eosinophilic deposits in the subepithelial layer (Figure 3) that stained positive with Congo red and Dylon (Figure 4). Furthermore all specimens showed retention of congophilic staining when treated with potassium permanganate, consistent with primary (AL) amyloid.

This was followed by biopsies of the stomach, duodenum and rectum, all of which were negative for amyloid. There was no Bence-Jones proteinuria, and serum or urine protein electrophoresis did not reveal any monoclonal band. Therefore, the patient was diagnosed with primary localized amyloidosis of the urinary bladder.

To treat residual amyloidosis of the bladder, we performed occlusive dressing therapy (ODT) using dimethyl sulfoxide (DMSO) every day. After percutaneous administration of DMSO, regression of the bladder lesions as well as improvement of gross hematuria were achieved. Five years after the initiation of treatment, cystoscopy and MRI showed no relapse of the mass-like lesion of the bladder wall. Currently, DMSO is administered every few days as maintenance therapy.

## Discussion

Amyloidosis is characterized by the extracellular deposition of proteins. Amyloidosis may be primary or secondary depending on whether it is due to underlying immune dyscrasia or secondary to a chronic inflammatory disorder. Although systemic amyloid can occur anywhere in the urinary tract, including the kidney, renal pelvis, ureter, urethra or corpora, primary localized amyloidosis of the urinary bladder is a rare urological disease [4-6].

Amyloidosis of the urinary bladder affects males and females equally in about the fifth and sixth decades of life. Previous studies have reported gross painless hematuria as the most common presentation and have stated that the remaining patients present with irritative voiding symptoms [4-6]. Primary amyloidosis of the urinary bladder assumes clinical importance because it clinically masquerades as a malignancy as in the present case.

Histological examination of the lesion is mandatory for diagnosis. Typically, primary amyloidosis deposits are superficially beneath the surface mucosa, sometimes extending into the superficial smooth muscle of the urinary bladder. In secondary amyloidosis, the amyloid tends to accumulate in the bladder vasculature thus explaining why secondary amyloidosis with diffuse bladder involvement has a high mortality of 30% with its potential for massive hemorrhage [7].

Management of primary amyloidosis of the urinary bladder is mainly transurethral resection of the bladder if it is localized. Medical treatments such as intravesical instillation of DMSO have also been tried with limited success.

DMSO is a dipolar solvent, which has a molecular weight of 78.13. DMSO has the action of penetrance acceleration, local anesthetic effect, sedative action and anti-inflammatory effect as pharmacological action. The penetrance action of DMSO is characteristic and it passes with a high concentration quickly through a skin barrier. Isobe [8] reported that when 90% DMSO solution was applied to human skin, it was identified in the blood 5 minutes later, and maximum blood concentration was obtained 4 to 6 hours later. In addition, this plateau was reported to last 36 to 72 hours [8]. The internal use, application to the skin and enema administration have been tried successfully since the efficacy of DMSO for amyloidosis was reported by Osserman *et al.* in 1976 [9].

**Figure 5:**
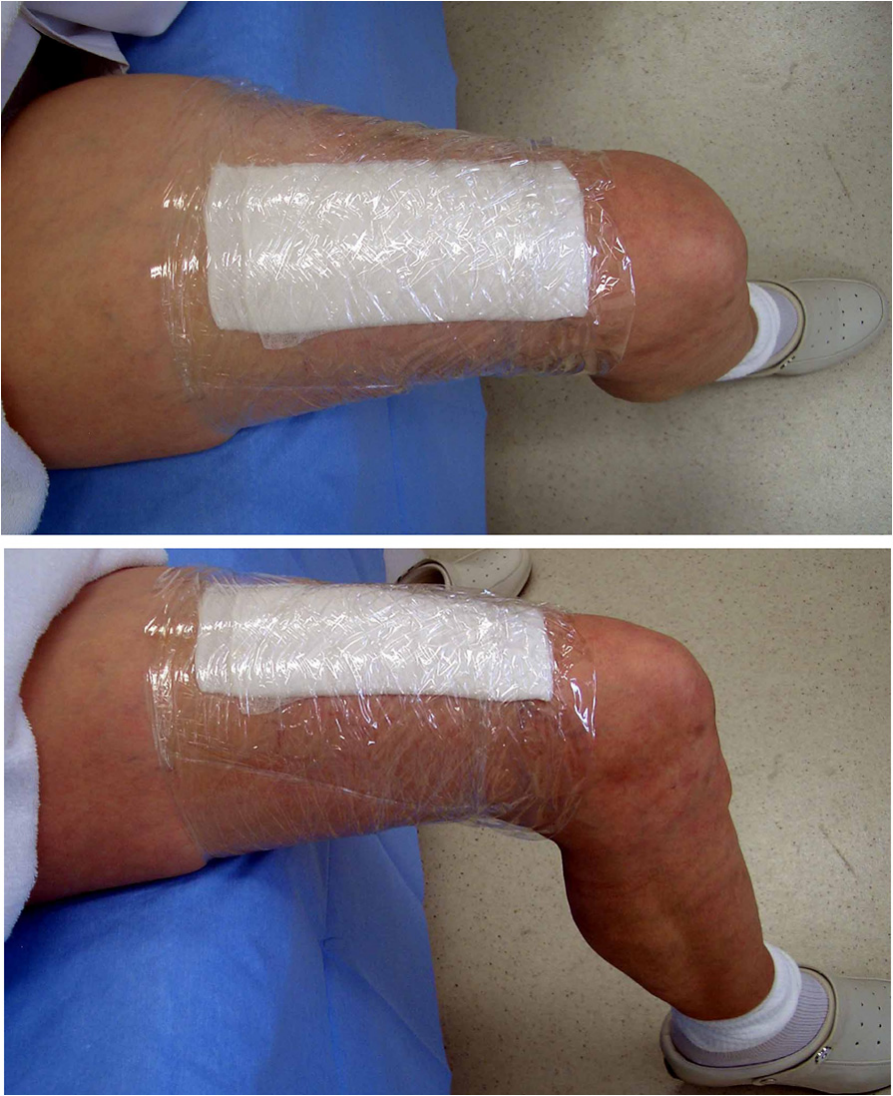
**Clinical photograph of the occlusive dressing therapy using dimethyl sulfoxide.** The gauze impregnated with 7mL of 50% dimethyl sulfoxide solution was attached to the skin of the patient’s thigh which was covered with cling wrap.

To the best of our knowledge, this is the first case in the English literature of localized amyloidosis of the urinary bladder effectively treated with ODT using DMSO. Previous Japanese literature has also reported the efficacy and tolerability of ODT using DMSO for amyloidosis of the urinary bladder [10-12]. In our case, in accordance with previous reports [10-12], the gauze of the ODT was dipped in a setup 7mL dose of 50% DMSO solution. The gauze impregnated with the solution was attached to the skin of the patient’s thigh, which was covered with cling wrap for about 2 hours every day (Figure 5).

The intravesical instillation of DMSO has already been established in the treatment of amyloidosis of the urinary bladder [4]. However, for this case, the cumbersome maneuver was considered to be a fault. The maneuver of ODT was simpler and easier than that of intravesical instillation, and ODT was advantageous in that the patient could herself make the ODT every day. In the present case, no severe side effects were observed and the treatment compliance of the case was good. Because there was no established theory about the duration of DMSO-ODT, the DMSO was administered every few days as maintenance therapy in our case. From now on we will extend intervals of administration gradually, monitoring for relapse of amyloidosis. There were less than 10 reported cases treated with ODT using DMSO for amyloidosis of the urinary bladder. Therefore, there is a need to accumulate data and demonstrate the optimum method of administration (concentration of solution, volume, application time) and the optimum duration of treatment.

In localized amyloidosis of the urinary tract, despite a few reports of recurrence, the prognosis is comparatively good. In addition, there were few cases of amyloidosis of the urinary bladder that required invasive surgical management such as cystectomy. Therefore, we think that conservative medical management such as ODT using DMSO should be tried first, leading to bladder preservation.

Invasive surgical management including cystectomy should be considered only if conservative management such as ODT using DMSO for several months does not induce a reduction of the bladder mass or improve gross hematuria.

## Conclusions

ODT using DMSO is efficacious and tolerable for amyloidosis of the urinary bladder. The maneuver of ODT was simpler and easier than that of intravesical instillation. However, invasive surgical management including cystectomy should be considered if conservative management is inefficacious.

## Consent

Written informed consent was obtained from the patient for publication of this case report and any accompanying images. A copy of the written consent is available for review by the Editor-in-Chief of this journal.

## Competing interests

The authors declare that they have no competing interests.

## Authors’ contributions

TY, SO and HM were involved in the initial writing of the manuscript. SO provided major editing changes. TY and HM were primarily involved in the care of the patient. SO and HM provided intellectual contributions to the content of the manuscript as well as editorial assistance. All authors have read and approved the final version of the manuscript.
